# Are We Scientific?

**Published:** 1917-07

**Authors:** George L. Servoss

**Affiliations:** Reno, Nevada, Editor “Western Medical Times.”


### Are We Scientific?

By GEORGE L. SERVOSS, M. D., Reno, Nevada,

Editor "Western Medical Times."

Reduced to the simplest possible term, science is the knowledge of a thing—a complete knowledge, or one as complete as it is possible to attain.

In our study and application of things therapeutic are we invariably scientific? Do we obtain anything like a complete knowledge of all the things pertaining to drugs and chemical and their uses? Is it not very frequently the case that, for one reason or another, we turn our backs on and will have nothing of this, that or the other drug, and for the simple reason that some supposed authority or board of censors has told us that such agent is either inactive or too active? Does it not very frequently occur that we never go farther than to read one side of the argument and thus do not avail ourselves of some drug or other remedial agent which may have been proved of value by someone else?

Not long ago a new edition of one of the leading text-books on materia medica and therapeutics came into our hands and in reviewing it we noticed that the author said, in rather an imperative manner, that the two alkaloids, aconitin and veratrine **must not** be administered internally—that they were too dangerous and too potent for any such application. Had we been unacquainted with these two agents we should undoubtedly have taken the word of the author as final law on the subject, but having employed both, **and without the least sign of deleterious effect,** over a term of more than a decade, we concluded that the writer had based his opinion upon the teachings of someone who, like himself, had never employed these alkaloids. He did not say that either alkaloid was inactive or that it would not give the effect of the parent drug, but based his instructions for non-use solely upon the question of too high potency. Elsewhere in his book he advised the use of atropine, an alkaloid every bit as potent as either of the ones which were consigned to the discard. The trouble was that this man, like many others, was not scientific—not wholly so. He had never employed either aconitin or veratrin and so, from a practical working standpoint, knew little or nothing of either. He did give the dosage of both, and as usual, with men of his sort, it was altogether too high. He did not, as do those who have employed these alkaloids successfully, and without untoward results, know that small and frequently repeated doses, not only give good results, but that such results are obtained without any bad conditions following.

Not long ago the drug crataegus was suggested to us for use in certain heart conditions. Desiring to know more about it than was contained in the information given us, we went to our text-books on materia medica originating from men of the regular school. After going through three of those written by teachers and accepted authorities on the subject of drugs and their applications we were no better off than we had been before picking up the first book. There was but one allusion made to the drug and that was simply a mere passing mention in the portion of the book devoted to therapeutics. Absolutely nothing was said in that part of the book given over to materia medica. Consequently, our knowledge was not increased by a single thought, even though we gave some time to our hunt for information. After exhausting all we could place our hands on, coming from writers of the regular school, we turned to a work by an eclectic and there we found several pages devoted to the drug in question and were able to obtain a very good knowledge of its actions and uses. Upon clinical application, we found that crataegus gave such results as were claimed for it by the eclectic writer. In view of this fact, are we of the regular school scientific therapeutists when we refuse the use of any such drug, simply because it originally came to us from a layman or from a school not our own?

We do not know why so little attention is paid to crataegus by the writers of the school. We have no information at hand relative to the composition of the drug and do not, consequently, know whether it carries a demonstrable active principle or not. We have an idea that it does not and that is the possible reason why it is not accepted by some of the regulars. But that it does the work expected of it, has been demonstrated by men both in and out of the regular school.

Cactus is another drug which is passed by by many of the regular writers, and for the reason that no active principle is demonstrable, and in spite of the fact that the clinician has found, in a practical way, that, in nervous heart manifestations, it does bring results, and good ones. Only recently have we had three cases of irregular heart action, without organic lesion, in which cactus has brought a return of the normal. In all of these instances no other agent was employed, as we desired to demonstrate, for our own satisfaction, whether or not the drug was efficient. Again have we had to go to the so-called irregular authorities for our information on the subject. The regular writers have either just given a mere mention of cactus or omitted it entirely from their books. Is this scientific? Does such omission lead up to a proper knowledge of the subject? Does it show us that the writers have given us information, based upon their own experience, or that they have relied upon others, equally without practical knowledge?

We have been told, time and again, that it is practically futile to exhibit the intestinal antiseptics and for the reason that the gut is so full of kinks, twists, turns and pockets, that no agent of this sort will render it anything like aseptic. For sake of argument, we will admit that, to some extent, this may all be very true, but those of us who have employed the antiseptics, know that we have gained results which no other agents have given us. We have seen a diarrhea cease as if by magic and with absolutely no recurrence. This was not the case when we depended upon opiates, which simply checked the flow, but did not have any effect upon the cause, than to possibly increase it. In intestinal fermentation, with stinking stools, we have seen the odor disappear completely, and very frequently within a very short time after a few full doses of the sulphocarbolates. We have seen fever, due to such fermentation, abate without the use of one single other remedy. It may be true that the entire bowel is not rendered aseptic, or nearly so by any such medication, but is it scientific to spread the information that the intestinal antiseptics are wholly useless, simply because of the supposed impossibility to render the whole of the gut clean? Is it not more scientific to base our ideas upon the knowledge of what we have gained in a clinical way, rather than upon theory?

We of the regular school have been too hide-bound in our ideas relative to drugs, their actions, uses and applications. We have formed too much of a habit of going about with our noses so elevated as to be unable to see that which has been at our feet. We have formed a mental corporosity which has interfered with our seeing our toes, and in consequence we have tripped over the unnoticed in a good many instances. In our treatment of things therapeutic we have relied upon the laboratory and not upon real common sense to too great an extent. We have been destructive, rather than constructive, both in our ideas and actions. We have been too prone to find fault without any real scientific foundation upon which to base such fault. We have been too prone to listen to others rather than to make investigations on our own initiative. In fact, in some quarters, the individual initiative would be wiped out completely and we would, if they would have their way, carry on our practice upon the basis of their thoughts, rather than upon our own. We would, in other words, be the "office boys" of the self-constituted authorities. We would do nothing unless they said so, and then only as they said.

It has been the lack of the scientific (complete) knowledge which has, within recent years, played havoc with the profession as a whole. We have been told we were know nothings until we have about concluded that such is the truth. That is, some of us have. We have, following the foot-steps of the self-constituted censors and authorities, gone on and on in our career of therapeutic destruction until now chaos reigns. That this is true is shown by the fact that one of our leading medical schools has determined to drop the department of materia medica and therapeutics from its curriculum, saying that nothing is of avail, or words to that effect, other than surgery. It tells us, in almost as many words, that drugs are of no avail and that our patient will either recover or die, no matter what effort may be made by us through the use of drugs. In other words, this school would destroy the very foundation of the practice of internal medicine, the relief of the sick. It is not a diagnosis of his condition that interests the sick individual, as a rule, but that something which will bring him back to normal, and in the quickest possible manner. He is not particularly interested in the etiologic factors of his disease, but in that which will wipe out the cause and make him a well man. He is not going to be satisfied with being told what ails him and then allowed to lie quietly and allow nature to take her course, good or bad. Not by any manner of means. He is going to insist upon attention from the **scientific** physician, the man who can not only make a diagnosis through recognition of the cause and pathology, but one who knows how to apply those remedies which will meet the indications in the case. He will have no use, **absolutely no use,** for the destructive physician, the man who would let him lie and die because of lack of proper therapeutic knowledge.

It is little wonder that the irregular practitioners are boring into our business. They "do things." They discover what ails their patient and then go ahead and give him all possible relief, and through such therapeutic channels as they have seen are really navigable. If one watches and compares them with men of our own school, it is readily seen that, case for case, their results in internal medicine equal, if not excel, our own. And the patient sees that they are making an endeavor to bring him relief and he is satisfied, even though they may fail, in some instances.

We will admit that we may be considered heretical in offering any such ideas as these, but the time has come when we must begin a reconstruction of things therapeutic. We have been destructive for too many years and with the result that we are no longer real healers of the sick. Through our destructive and unscientific actions we have given every irregular just the opening desired, that of demonstrating that we are inherently weak, that we are only half taught, and very frequently that half poorly. We have paved the way for the success of the Christian Scientist, osteopath, chiropractor and all other sects and cults, and all for the reason that we have been half-baked in the **real** art of the doctor, the proper treatment of the sick. We **are not** scientific, when it comes to things therapeutic, because we do not possess a complete knowledge of those things which we should employ.

---

**Ether-Chloroform Mixtures.** W. J. McCardie, Brit. Med. Jour., April 21, experimented with various mixture to avoid the irritating effects of ether, soldiers being especially to be considered on account of exposure resulting in pharyngitis, and excessive smoking; and, on the other hand, the depressing effects of chloroform. The mixtures used varied from 4 parts of ether to 1 of chloroform up to 32 of ether to 1 of chloroform (Note the influence of the apothecary's system) and he found the happy mean to be 16 of ether to 1 of chloroform. He gives 1/6 gr. (1 c.g.) of morphine and 1/100 (about 2/3 m.g.) of atropine before the general anaesthetic.

---

**Rarity of Conjugal Phthisis.** Maurice Fischberg, Am. Jour. Med. Sci., page 395 Vol. 153, in 170 families in which husband or wife was known to be tuberculous, found only 5 cases in which both were tuberculous—2.9%. As approximately 10% of all persons develop tuberculosis of serious degree from which both were tuberculous—2.9%. (As approximately 10% of the mates of those already infected would become so without any special influence from the domestic fomes. Hence the predisposition seems to be about three times as great as for the population at large.)

---

**Double Radial Artery.** This rare anomaly is described by Edward Faucett of Bristol, Eng., Brit. Med. Jour., Mch. 17. It was associated, as it frequently is, with three heads of the biceps, the brachial branching high.

